# Development of a Genetic Map for Onion (*Allium cepa* L.) Using Reference-Free Genotyping-by-Sequencing and SNP Assays

**DOI:** 10.3389/fpls.2017.01606

**Published:** 2017-09-14

**Authors:** Jinkwan Jo, Preethi M. Purushotham, Koeun Han, Heung-Ryul Lee, Gyoungju Nah, Byoung-Cheorl Kang

**Affiliations:** ^1^Department of Plant Science, Plant Genomics and Breeding Institute, Vegetable Breeding Research Center, College of Agriculture and Life Sciences, Seoul National University Seoul, South Korea; ^2^Biotechnology Institute, Nongwoo Bio Co., Ltd. Yeoju, South Korea; ^3^National Instrumentation Center for Environmental Management, Seoul National University Seoul, South Korea

**Keywords:** onion, genotyping-by-sequencing, linkage map, Fluidigm, single nucleotide polymorphism (SNP)

## Abstract

Single nucleotide polymorphisms (SNPs) play important roles as molecular markers in plant genomics and breeding studies. Although onion (*Allium cepa* L.) is an important crop globally, relatively few molecular marker resources have been reported due to its large genome and high heterozygosity. Genotyping-by-sequencing (GBS) offers a greater degree of complexity reduction followed by concurrent SNP discovery and genotyping for species with complex genomes. In this study, GBS was employed for SNP mining in onion, which currently lacks a reference genome. A segregating F_2_ population, derived from a cross between ‘NW-001’ and ‘NW-002,’ as well as multiple parental lines were used for GBS analysis. A total of 56.15 Gbp of raw sequence data were generated and 1,851,428 SNPs were identified from the *de novo* assembled contigs. Stringent filtering resulted in 10,091 high-fidelity SNP markers. Robust SNPs that satisfied the segregation ratio criteria and with even distribution in the mapping population were used to construct an onion genetic map. The final map contained eight linkage groups and spanned a genetic length of 1,383 centiMorgans (cM), with an average marker interval of 8.08 cM. These robust SNPs were further analyzed using the high-throughput Fluidigm platform for marker validation. This is the first study in onion to develop genome-wide SNPs using GBS. The resulting SNP markers and developed linkage map will be valuable tools for genetic mapping of important agronomic traits and marker-assisted selection in onion breeding programs.

## Introduction

Onion (*Allium cepa* L.) is one of the most widely cultivated vegetable crops grown in tropical, temperate and boreal regions around the world. The economic value of onion derives from its culinary applications, nutritional benefits, and health-promoting properties. Onions are grown throughout the year in 175 countries on around 6.6 million hectares, yielding a production of 742.51 million tons (FAOSTAT, 2014). Despite its significance, genetic and genomic research on onion has been limited due to its biennial life cycle, high inbreeding depression, cross-pollinating nature, and large genome size (McCallum et al., 2006; Duangjit et al., 2013). Enhancing the onion genomic resources is critical to promote the conservation of its germplasm and to increase crop quality, productivity, adaptability, and resistance to disease (McCallum, 2007).

Single nucleotide polymorphisms (SNPs) are the most common and preferred genetic markers, favored for their high abundance, even distribution, and strong association to traits of interest (Hayward et al., 2012). SNPs can also be converted into molecular markers suitable for high-throughput genotyping assays and thus, SNP discovery and genotyping have increasingly gained the attention of researchers (Varshney et al., 2009). The development of SNP markers offers exciting opportunities for onion breeding programs; however, it has always been a formidable task in this species due to two major challenges. First, the large genome of 16 Gbp, which is 100 times larger than Arabidopsis and 18 times than the tomato genome. The second challenge is that onion often contains high levels of heterozygosity (King et al., 1998). Attempts to sequence the onion genome have benefitted from a few low- to medium-throughput platforms, such as studies using restriction fragment length polymorphisms (RFLPs; King et al., 1998; McCallum et al., 2001), amplified fragment length polymorphisms (AFLPs; Ohara et al., 2005), and simple sequence repeats (Baldwin et al., 2012). With the advent of next-generation sequencing (NGS) technologies, SNP markers were generated from the expressed regions of the genome of an inbred onion population and used to construct a genetic map (Duangjit et al., 2013). Similarly, a study used an interspecific F_1_ hybrid and a set of onion cultivars to develop transcriptome-derived SNP markers and a genetic map, which were used to identify quantitative trait locus for resistance to Botrytis leaf blight (Scholten et al., 2016). Large numbers of new molecular markers are still required to construct saturated consensus genetic linkage maps for onion and to significantly improve marker-assisted selection for both research and breeding objectives.

Genotyping-by-sequencing (GBS) is a simple yet robust approach for reducing genomic complexity to perform high-throughput genotyping of crops with large and complex genomes (Elshire et al., 2011; Baldwin et al., 2012). GBS has the advantages of being low cost, no reference sequence limits, reduced sample handling, and simple scalability (Davey et al., 2011). Although GBS efficiently generates SNP markers, it has some potential drawbacks, such as genotyping errors, missing data, and the under-calling of heterozygous sites (Swarts et al., 2014). However, GBS has been successfully applied for SNP discovery and genotyping in plant species with complex genomes, such as barley, maize, soybean, wheat, and chickpea (Elshire et al., 2011; Romay et al., 2013; Iquira et al., 2015; Jaganathan et al., 2015; Li et al., 2015).

In this study, we developed a genome-wide SNP resource in onion using a GBS *de novo* approach on an F_2_ population. Further, the filtered robust SNPs were used to construct a genetic map and were validated using Fluidigm genotyping assay. To our knowledge, this is the first genetic map based on genome wide SNP calling using GBS to date for onion. The use of GBS combined with SNP validation assays have generated robust molecular markers for dissecting genetic architecture of agronomical important traits in onion breeding programs.

## Materials and Methods

### Plant Materials and DNA Extraction

Two onion inbred lines of ‘NW-001’ and ‘NW-002’ were crossed at Nongwoo Bio Co., Ltd., South Korea, to produce an F_1_ population. The F_1_ hybrid plants were self-pollinated to produce an F_2_ segregating population. Among these, 92 F_2_ individuals and two plants from each parental line (M1 and M2 from NW-001 and P1 and P2 from NW-002) were used for the GBS analysis. Total genomic DNA (gDNA) was prepared from leaf tissue using the cetyltrimethylammonium bromide (CTAB) method (Doyle and Doyle, 1987). The quality and quantity of the gDNA were evaluated using a Take3 Micro-Volume plate (BioTek Instruments, Inc., Winooski, VT, United States) in an ‘Epoch’ spectrophotometer (BioTek Instruments, Inc.). The samples were then diluted to a concentration of 50 ng/μL and stored at -20°C until use.

### Library Construction and Illumina Sequencing

The GBS library was prepared using the Illumina TruSeq protocol as described by Truong et al. (2012). Briefly, 400 ng gDNA from the plant samples was digested with *Pst*I and *Mse*I. The resulting fragments from all samples were ligated to a pair of enzyme-specific adapters with different barcodes assigned to each sample. The samples were then combined into pools and amplified by 50 cycles of PCR to generate the GBS library. The library was added to a Bioanalyzer DNA 1000 Chip (Agilent Technologies, Santa Clara, CA, United States) and its quality was checked. The library was then pooled and sequenced using a HiSeq4000 (Illumina, Inc., San Diego, CA, United States) at Macrogen Co., Ltd. (Seoul, South Korea). The raw data were produced as fastq files.

### Assembly and SNP Calling

Raw reads were demultiplexed in accordance with the individual sample barcodes. The adapters and barcodes were trimmed and the reads were *de novo* assembled using CLC Genomics Workbench version 8.0 (CLC Bio, Aarhus, Denmark). The assembled contigs were trimmed to 140–151 bp. The filtered raw reads were mapped to the assembled contigs using Burrows-Wheeler Aligner (BWA) version 0.7.12 (Li and Durbin, 2010). Picard Tools version 1.119 and SAMtools version 1.1 were used for read grouping and sorting (Li et al., 2009). For genome-wide SNP calling, Genome Analysis Toolkit (GATK) Unified Genotyper version 3.3 was applied. High-quality SNPs with a QUAL value larger than 30 and a minimum depth of 3 were selected for further analysis.

Single nucleotide polymorphisms were then selected based on their heterozygosity and parent call. The parent call step was performed with merged duplicates of the parental genotypes (M1^1^, M1^2^-M1 and P1^1^, P1^2^-P1) along with each of parental siblings (M2 and P2) in the sequencing pool. Each of the parent genotypes were tagged with three different barcodes and thereby, the combined sequencing depths of the parents were increased threefold relative to the F_2_ individuals. The SNP calls were presented as: ‘0/0’, alleles with the same SNP; ‘1/1’, alleles with a different SNP; ‘0/1’, heterozygous alleles; and ‘./.’, no call. SNP-flanked contigs were analyzed for amenable markers using the D3 Assay Design program (Fluidigm Corporation, San Francisco, CA, United States). Amenable SNP markers were validated using a Fluidigm assay.

### Marker Genotyping

Genotyping was performed using the Fluidigm EP1^TM^ system (Fluidigm Corporation). A pre-amplification step was performed with combinations of a specific-target amplification primer and a locus-specific primer. The pre-amplified products were then diluted in distilled water and subjected to a second round of PCR amplification using a set of fluorescently labeled allele-specific primers. The sequences of all primers used for the Fluidigm assay are presented in Supplementary Table S1. SNPs were then called according to the manufacturer’s protocol using pre-defined algorithms in the Fludigim SNP Genotyping Analysis software.

### Genetic Map Construction

The genetic map construction was performed as previously described by Schiex and Gaspin (1997). The GBS obtained SNPs that fit the 1:2:1 segregation ratio (*p-*value > 0.01) and one locus was selected per contig consisting of multiple SNPs. The linkage analysis was performed using CarthaGene software with the selected GBS SNPs and Fluidigm markers. To further confirm the marker robustness, the LOD threshold was set at 3.4 and the maximum distance was set at 50 centiMorgans (cM). Marker loci were allotted into eight linkage groups based on their distributions. The genetic distance between the markers was estimated in cM using the Kosambi mapping function (Kosambi, 1943). The resulting genetic linkage map was drawn using MapChart 2.3 software (Voorrips, 2002).

## Results

### GBS Library Construction and Sequencing

Our Illumina sequencing generated approximately 794,687,530 single-end reads totaling 56.1 Gbp (**Table 1**). This high number of raw sequencing reads across the collection of samples reflected reduced levels of contamination and unexpectedly low sequence repeats. Upon cleaning the raw data, we obtained 356,820,838 reads, which were subjected to further trimming and demultiplexing. A total of 1,300,981 contigs, covering 222 Mbp with an N50 value of 144 bp, were *de novo* assembled to generate the reference genome. The minimum and maximum lengths of the contigs were 100 and 2,806 bp respectively, with an average of 150 bp. Most of the contigs (83%) were either 144 or 145 bp (Supplementary Figure S1). The GC content was in the range of 40–45% and the over-represented sequences were minimal.

**Table 1:** Summary of GBS data for the mapping population.

Summary of Illumina sequencing	
Total number of bases	56.15 Gbp
Total number of reads	794,687,530
Trimmed reads	742,403,872 (93.4%)
Demultiplexed reads	356,820,838 (44.9%)
***De novo* assembled reference contigs**	
N50	144 bp
Average	150 bp
Total contigs	1,300,981 (222 Mbp)
Trimmed contigs (140–151 bp)	509,546 (33%)


### SNP Calling and Filtering

We selected 509,546 (33%) contigs from the *de novo* assembly with a length of 140–151 bp for SNP calling based on Illumina maximum read length. The complete sequences of these functional contigs is made available online with DOI: 10.6084/m9.figshare.5363338. In total, 1,851,428 SNPs were predicted from 201,274 assembled contigs. We then filtered 1,399,567 high-quality SNPs from 135,813 contigs based on their mapping score (**Table 2**). After the quality and depth filtering, SNPs with missing data, missing parental genotypes, or shared heterozygous genotypes were removed.

**Table 2:** Filtering steps to identify robust SNPs in *de novo* assembled contigs.

Step description	Number of SNPs	Number of SNP carrying contigs
Raw SNPs	1,851,428	201,274
Qual 30-filtered SNP	1,399,567	135,813
SNP of heterozygotes^a^	82,920	13,792
SNP of no polymorphic loci^b^	188,143	8,900
SNP of no call^c^	1,118,413	132,664
Polymorphic SNP	10,091	3,846
Called SNP for >60% samples	971	528
1:2:1 segregation	202	103
D3-validated SNP markers	122	84


*^a^At least one parent has ‘0/1’ called data. ^b^Both parents have the same call, excluding‘0/1’. ^c^At least one parent has a no-call, ‘./.’.*

The ‘heterozygosity call’ and ‘no call,’ where one or both parents categorized as ‘0/1’ or ‘./.’ resulted in 82,920 SNPs and 1,118,413 SNPs, respectively. Following this, the ‘no polymorphism call,’ where both parents were classified as either ‘0/0’ or ‘1/1’, was assigned to 188,143 SNPs and eliminated. The remaining 10,092 SNPs were finalized from the parental call and were compared between the genotypes of 90 individuals from the F_2_ population (Supplementary Table S2). Based on the presumed genome size, the frequency of unfiltered SNPs was one SNP in every 8.6 kb, whereas the frequency of the filtered SNPs was one SNP every 1,586 kb, indicating the stringency applied in the calling of reliable SNPs. Furthermore, SNPs with <60% called genotypes in the F_2_ population were excluded, resulting in 971 SNPs. Amenable SNP markers were selected for map construction and Fluidigm genotyping based on segregation ratios and the D3 assay, resulting in 202 and 122 robust SNPs, respectively.

### Fluidigm Genotyping

The selected amenable SNPs were developed and tested for molecular marker conversion using the Fluidigm EP1^TM^ genotyping system (**Table 3** and Supplementary Table S3). The average and maximum matching rates of the SNP markers with validated molecular markers were found to be 74 and 100%. Among the selected 96 SNP markers from the D3 assay, 66 SNP markers were validated with greater than the average matching rate of 83%. Of these, 57 markers had a 1:2:1 segregation ratio, giving a success rate of 59%. The Fluidigm protocol ensured that one SNP from every contig was considered for the construction of molecular markers. Upon failure, an alternative and yet still efficient SNP from the same contig was then assessed to retain the maximum possible number of molecular markers for genome construction.

**Table 3:** Fluidigm assay output.

Used SNP markers for genotyping	96
Average matching rate	74%
SNPs with more than the average matching rate	66
Markers with 1:2:1 segregation	57
Success rate	59%
Maximum matching rate	100%
Average matching rate (57 SNP markers)	83%


### Linkage Map Construction

**FIGURE 1:**
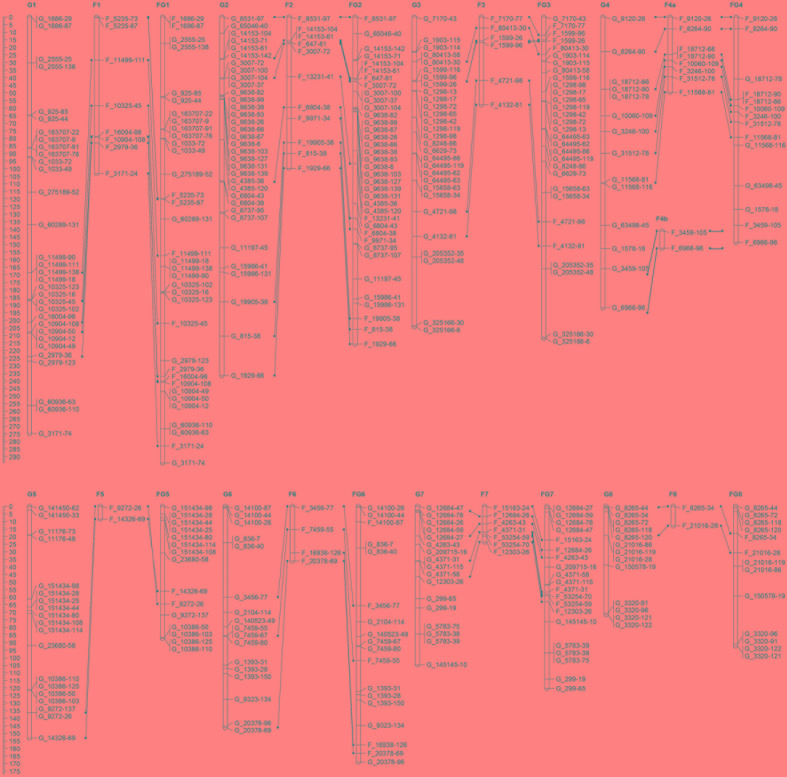
Distribution of high-quality molecular markers on eight linkage groups of the onion F_2_ population. Genetic maps of single nucleotide polymorphisms (SNPs) from genotyping-by-sequencing (G; left) and Fluidigm molecular markers (F; center), with a consolidated map (FG; right). Genetic distances are in centiMorgans (cM) and lines correspond to relative marker positions.

A set of 202 SNPs satisfying all filtering procedures were used for the construction of a GBS-based linkage map (‘G’ linkage groups). The Fluidigm-validated markers were also applied to map construction (‘F’ linkage groups), the results of which were then integrated with the GBS markers to form a consolidated map of “FG” linkage groups. Once the frame map was constructed, the validated SNPs were anchored to the linkage groups sequentially until all markers were successfully assigned, creating the final map. All three maps consisted of eight linkage groups (**Figure 1**). The GBS linkage map comprised 175 SNP markers and spanned a total of 1,383 cM, with an average marker distance of 8.08 cM (**Table 4**). On average, one linkage group contained 21.87 markers spanning a length of 172.87 cM. The largest linkage group, G1, harbored 32 markers covering 274.8 cM with an average of only 8.6 cM between adjacent markers. The smallest linkage group, G8, contained 13 markers, with a length of 71.7 cM and an average inter-marker distance of 5.52 cM. The percentage of polymorphism ranged from 19% in G2 to 7% in G8. The consolidated linkage map, containing markers mapped from both GBS and Fluidigm, comprised 182 markers covering a total length of 1339.5 cM (**Table 5**). The combined linkage group lengths were variable; FG1 was the largest, spanning 294.2 cM with 35 markers, while FG5 covering the shortest distance, 87.3 cM, with 15 markers. The average marker distance was 7.53 cM, with the maximum gap ranging from 20.6 cM in FG7 to 45.3 cM in FG3. On average, each FG contained 22.75 markers covering 167.43 cM. Though FG1 was the largest among the linkage groups in the consolidated map, FG2 harbored the highest number of markers with a virtually even marker distribution across its length. A detailed description of all linkage groups with marker names and cM positions is shown in Supplementary Table S4.

**Table 4:** Summary of the onion genetic map constructed using SNP markers.

Linkage group ID	Total SNPs	Genetic distance (cM)	Average marker interval (cM)	Maximum gap (cM)	Polymorphism rate (%)
G1	32	274.8	8.59	34.2	18
G2	34	236.5	6.96	27.2	19
G3	29	204.3	7.04	45.4	17
G4	14	191.7	13.69	27.4	8
G5	19	153.1	8.06	29.1	11
G6	17	146.2	8.60	36.1	10
G7	17	104.7	6.16	20.4	10
G8	13	71.7	5.52	31.6	7
Total	175	1383	-	-	100
Average	21.875	172.875	8.08	31.43	12.5


**Table 5:** Summary of the onion genetic map constructed using GBS and Fluidigm markers.

Linkage group ID	Total SNPs	Genetic distance (cM)	Average marker interval (cM)	Maximum gap (cM)	Polymorphism rate (%)
FG1	35	294.2	8.41	34.9	19
FG2	37	215.8	5.83	26.4	20
FG3	30	211.9	7.06	45.3	16
FG4	14	148.7	10.62	26.7	8
FG5	15	87.3	5.82	25.3	8
FG6	18	169	9.39	38.1	10
FG7	20	120.4	6.02	20.6	11
FG8	13	92.2	7.09	31.7	7
Total	182	1339.5	-	-	100
Average	22.75	167.4375	7.53	31.13	12.5


## Discussion

Genotyping-by-sequencing is a robust approach that uses enzyme-based genome complexity reduction coupled with barcoded DNA adapters to produce multiplexed sample libraries (Elshire et al., 2011). GBS has been increasingly used in genetic and genomic studies in a wide range of crop plants (Varshney et al., 2009; Deschamps et al., 2012; Poland and Rife, 2012). Although onion is among the most extensively cultivated and traded vegetable crops, marker resources are highly limited (McCallum et al., 2006), so we set out to use GBS to discover genome-wide SNPs in onion in a cost- and time-efficient manner.

Due to the heterozygosity of the onion parental lines, it was necessary to include additional replicates of the parents when sequencing the pooled GBS library; hence, we sequenced the GBS libraries with replicates of the parental genotypes (M1, M2, P1, and P2) to obtain homozygous SNP calls. We selected the *Pst*I-*Mse*I enzyme combination, which targets methylation-sensitive regions of the genome and led to a sufficient read depth to perform SNP calling. Only 33.1% of the trimmed contigs were aligned using the reads, possibly because of the stringent parameters applied by the BWA aligner to minimize multiple mapping. A similar limitation was also reported using the same tool for genome-wide SNP discovery in pepper (Taranto et al., 2016).

We used GATK for SNP calling, which filled the specifications required for SNP discovery from the trimmed reads in the absence of a reference genome sequence (McKenna et al., 2010; DePristo et al., 2011). Using this tool, we applied efficient filtering criteria to eliminate low-quality and heterozygous SNPs. Regardless of the complexities, 1,399,567 SNPs were identified in this study after the initial quality check. The average SNP frequency, one SNP per 12 kb of genome length, was higher than the previously reported values from transcriptomes, which found SNPs every 243 and 790 kb, respectively (Duangjit et al., 2013; Scholten et al., 2016). Restricting SNP development to the expressed portion of the genome limits genome coverage and potentially the density of SNP markers (Trebbi et al., 2011). We speculate that characterizing intron-spanning SNPs maximizes the probability of finding efficient molecular markers. The high frequency of SNPs retained after the initial quality check in our study indicates the importance of generating genome-wide SNPs, which, critically, include markers from regulatory regions as well as transcribed regions.

A major challenge encountered when using GBS methods is the complexity of aligning true alleles of each single locus in complex, heterozygous genomes such as onion (He et al., 2014). In addition, information on the levels of heterozygosity within a selected population would be of value for elucidating the underlying population structure and for future estimations of genetic gain (Baldwin et al., 2012). Our approach and selected tools have effectively calculated the heterozygosity and excluded just 82,920 SNPs or 5.9% of the quality filtered SNPs. This value is lower than those previously reported from transcriptome data, which consisted of 12.68% heterozygous SNPs (8,329 of the 65,675 SNPs; Duangjit et al., 2013). This unexpectedly lower frequency of heterozygous calls could be ascribed to the inbred nature of our samples or to bias arising from our relatively small sample size. The notable markers that remained were further filtered; however, successive SNP calling based on the parent call, segregation ratio, and missing data resulted in only 202 robust SNPs, of which 122 were considered amenable for Fluidigm genotyping. The number of SNPs identified in this study was limited by the capture of a reduced portion of the genome following the combination of the two enzymes and stringent SNP calling by GATK. Although additional SNPs could have been identified using imputation algorithms for missing data, we did not apply that procedure in favor of identifying reproducible and reliable SNP markers. Nevertheless, multiple GBS libraries generated using different combinations of enzymes or the use of multiple cutter enzymes will enlarge the sequencing pools and will thereby enable the capture of important genomic regions. These modifications to the protocol could be implemented in the near future to increase the density of identified robust SNPs.

Linkage groups were constructed and ordered based on their recombination frequencies and identified molecular markers. Despite the high stringency applied for the linkage analysis, we were able to construct linkage maps with the expected number of linkage groups, corresponding to the chromosome number of onion (*n* = 8). The linkage map was constructed using 175 SNP markers, which comprised only 1.73% of the SNPs from the parent call due to the exclusion of SNPs with missing data and segregation distortion. This percentage was similar to the amounts used in other GBS-based linkage mapping studies in apple (0.9% of identified SNPs) and sunflower (1.7% of unfiltered SNPs) (Gardner et al., 2014; Celik et al., 2016). The SNP linkage map spanned 1,383 cM, which is longer than the RFLP and AFLP maps of 1,064, 947, and 886 cM obtained previously (King et al., 1998; Ohara et al., 2005; Galván et al., 2011). Although the linkage map constructed from the previous transcriptome data appears to be densely covered by 479 SNPs (Duangjit et al., 2013), it should be noted that 140 EST markers from a former study (Martin et al., 2005) were included along with their newly identified SNPs. Moreover, it would be highly laborious to predict the correct genotypes using closely linked molecular markers with a high linkage disequilibrium that may be partially redundant (Scheben et al., 2017). The limited yet high-quality SNPs used in the construction of our linkage map will serve as an efficient genomic resource in onion marker-assisted selection; however, development of further bi-parental populations to increase the sample size for GBS-based SNP calling might increase the map density and serve as a more comprehensive reference for onion.

It is essential to validate SNP markers developed for crop improvement, especially in genomes of huge size and with highly repetitive sequences (Rasheed et al., 2016). In total, 96 SNP markers were selected as amenable and were validated using the Fluidigm assay. The average and maximum matching rates of the GBS-Fluidigm genotypes were 74 and 100%, respectively. A total of 66 SNPs scored more than the average matching and the success rate was estimated to be 59%. These matching rates are comparable to those previously reported for onion cultivars (74%) (Duangjit et al., 2013) and for *Lilium* (76%), an outcrossing species that also possesses a large genome (Shahin et al., 2012). With the validation of these SNP markers, the number of publicly available SNP markers for onion has increased: 43 SNP markers were validated in Martin et al. (2005), with a further 93 and 930 markers developed in 2012 (Baldwin et al., 2012; Duangjit et al., 2013).

## Conclusion

We obtained GBS derived SNP markers from a segregating F_2_ onion population in the absence of reference genome. Assembled filtering procedures and quality checks resulted in high fidelity SNPs which were subsequently applied for genetic map construction and Fluidigm validation. Thus, our study is a valuable addition to the present genomic resources of onion and these molecular markers act as valuable tools for cultivar identification, determining genetic diversity and relatedness among cultivars, and testing the authenticity and purity of onion inbred and hybrid lines. They can also be applied to effectively identify numerous gene conversions and crossovers. Furthermore, these SNP markers and the genetic map might be used as an anchoring scaffold for the physical mapping of genes upon the development of a reference genome sequence in the future. Ultimately, SNP markers from various studies could be combined to secure a consensus map of onion (McCallum et al., 2012), which would be highly valuable for the onion breeding community, enabling association studies, genetic diagnosis, analysis of quantitative trait loci, genomic selection, and efficient marker-assisted selection in onion.

## Author Contributions

B-CK, JJ, and KH conceived and designed the study. B-CK provided advice on the experimental design. JJ, KH, GN, and H-RL performed experiments. JJ, KH, and PP analyzed the data. PP and JJ wrote the manuscript. All authors have read and approved the final manuscript.

## Conflict of Interest Statement

The authors declare that the research was conducted in the absence of any commercial or financial relationships that could be construed as a potential conflict of interest.
