# Supplementary figures and images for: Development of a Genetic Map for Onion (Allium cepa L.) Using Reference-Free Genotyping-by-Sequencing and SNP Assays
Source: Front Plant Sci. 2017 Sep 14;8:1606. doi: 10.3389/fpls.2017.01606 (PMC5604068; doi:10.3389/fpls.2017.01606)

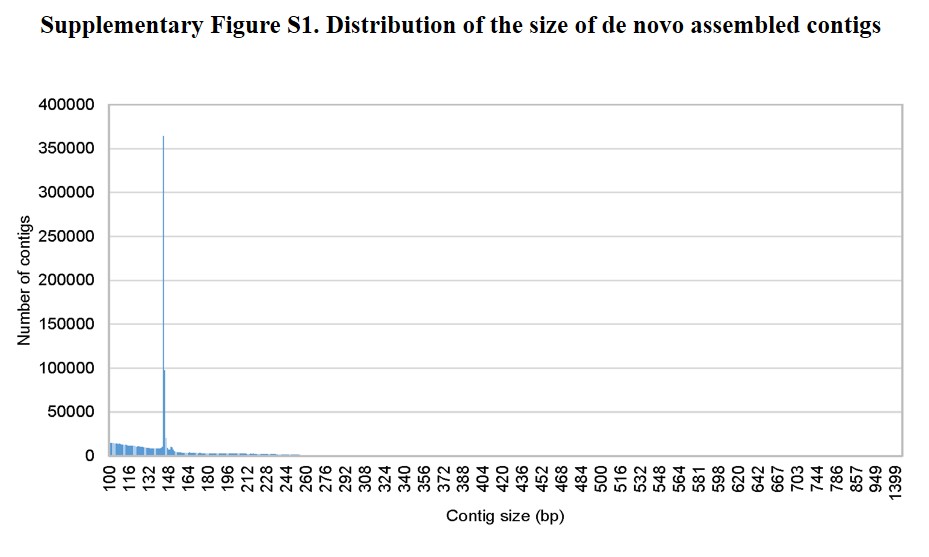

Supplement: Supplementary file 5 [file Image_1.JPEG]
